# Surgical strategy for coronary artery aneurysms

**DOI:** 10.1186/1749-8090-8-S1-O180

**Published:** 2013-09-11

**Authors:** M Hibino, K Tajima, K Uchida, H Munakata, K Fujii, W Kato, Y Takami, Y Sakai

**Affiliations:** 1 Cardiovascular Surgery, Nagoya Daini Red Cross Hospital, Nagoya, Japan

## Backgrounds

Coronary artery aneurysms (CAA) are relatively rare diseases caused by kinds of backgrounds such as atherosclerosis and connective tissue disorders, and the surgical strategy of them is still controversial. We have performed aneurysmectomy when the diameter is more than 10mm or CABG when the stenosis exists around the CAA. In this study, we evaluated our result of the strategy.

## Methods

Between 2002 and 2012, 10 patients (7 male, 68.7±13.0 yr) with CAA (6 LMT, 3 LAD, 1 RCA) had cardiac operations in our hospital. The mean size of the CAA was 20.2 (7 to 51mm). 7 CAA were saccular aneurysms, 2 were due to BWG syndrome and post PCI complication, respectively.

## Results

6 patients with more than 10mm CAA received aneurysmectomy and 6 patients with stenosis received CABG to the distal artery of CAA. Other concomitant procedures included MVP and AVR. The operative complications included 2 branch artery occlusions, but 1 was protected by CABG. There were no hospital death, and no CAA related event in 2.4±2.5 follow-up. In the postoperative CAG, there were no residual aneurysms after aneurysmectomy.

## Conclusions

Our surgical strategy for CAA was feasible. We need to pay attention to the branch occlusion in case of aneurysmectomy.

